# The Association Between Diet Quality and Glycemic Outcomes Among People with Type 1 Diabetes

**DOI:** 10.1016/j.cdnut.2024.102146

**Published:** 2024-03-26

**Authors:** Melanie B Gillingham, Martin Chase Marak, Michael C Riddell, Peter Calhoun, Robin L Gal, Susana R Patton, Peter G Jacobs, Jessica R Castle, Mark A Clements, Francis J Doyle, Michael R Rickels, Corby K Martin

**Affiliations:** 1 Department of Molecular and Medical Genetics, Oregon Health and Science University, Portland, OR, United States; 2 Jaeb Center for Health Research, Tampa, FL, United States; 3 School of Kinesiology and Health Science, Muscle Health Research Centre, York University, Toronto, Canada; 4 Center for Healthcare Delivery Science, Nemours Children’s Health, Jacksonville, FL, United States; 5 Department of Biomedical Engineering, Oregon Health and Science University, Portland, OR, United States; 6 School of Medicine, Division of Endocrinology, Diabetes and Clinical Nutrition, Harold Schnitzer Diabetes Health Center, Oregon Health and Science University, Portland, OR, United States; 7 Department of Pediatrics, Endocrine/Diabetes Clinical Research, Children’s Mercy Hospital, Kansas City, MO, United States; 8 Harvard John A. Paulson School of Engineering and Applied Sciences, Harvard University, Cambridge, MA, United States; 9 Division of Endocrinology, Diabetes & Metabolism, University of Pennsylvania Perelman School of Medicine, Philadelphia, PA, United States; 10 Pennington Biomedical Research Center, Louisiana State University, Baton Rouge, LA, United States

**Keywords:** Type 1 diabetes, diet quality, glycemia, time in range, Healthy Eating Index, humans, exercise

## Abstract

**Background:**

The amount and type of food consumed impacts the glycemic response and insulin needs of people with type 1 diabetes mellitus (T1DM). Daily variability in consumption, reflected in diet quality, may acutely impact glycemic levels and insulin needs.

**Objective:**

Type 1 Diabetes Exercise Initiative (T1DEXI) data were examined to evaluate the impact of daily diet quality on near-term glycemic control and interaction with exercise.

**Methods:**

Using the Remote Food Photography Method, ≤8 d of dietary intake data were analyzed per participant. Diet quality was quantified with the Healthy Eating Index-2015 (HEI), where a score of 100 indicates the highest-quality diet. Each participant day was classified as low HEI (≤57) or high HEI (>57) based on the mean of nationally reported HEI data. Within participants, the relationship between diet quality and subsequent glycemia measured by continuous glucose monitoring (CGM) and total insulin dose usage was evaluated using a paired *t*-test and robust regression models.

**Results:**

Two hundred twenty-three adults (76% female) with mean ± SD age, HbA1c, and body mass index (BMI) of 37 ± 14 y, 6.6% ± 0.7%, and 25.1 ± 3.6 kg/m^2^, respectively, were included in these analyses. The mean HEI score was 56 across all participant days. On high HEI days (mean, 66 ± 4) compared with low HEI days (mean, 47 ± 5), total time in range (70–180 mg/dL) was greater (77.2% ± 14% compared with 75.7% ± 14%, respectively, *P* = 0.01), whereas time above 180 mg/dL (19% ± 14% compared with 21% ± 15%, respectively, *P* = 0.004), mean glucose (143 ± 22 compared with 145 ± 22 mg/dL, respectively, *P* = 0.02), and total daily insulin dose (0.52 ± 0.18 compared with 0.54 ± 0.18 U/kg/d, respectively, *P* = 0.009) were lower. The interaction between diet quality and exercise on glycemia was not significant.

**Conclusions:**

Higher HEI scores correlated with improved glycemia and lower insulin needs, although the impact of diet quality was modest and smaller than the previously reported impact of exercise.

## Introduction

Nutrition plays a critical role in the daily management of type 1 diabetes. Persons with type 1 diabetes mellitus (T1DM) are advised to count carbohydrate intake, adjust bolus insulin delivery to food intake, and consume an overall healthy, high-quality diet. Multiple studies have demonstrated not just the carbohydrate-to-insulin ratio, but the type of foods that impact glycemic control. Consuming a high-quality diet with more fiber, fruits, vegetables, and a lower glycemic index have all been associated with improved HbA1c [1,2]. However, following this type of dietary pattern is challenging for individuals to maintain for numerous reasons. First, it requires meal planning, food preparation, time, access, and the ability to acquire healthy foods. Second, it entails resisting highly palatable foods, many of which are readily available and affordable. Many people, including those with T1DM, struggle to consistently eat a high-quality diet, and diet quality varies day-to-day [3]. The impact of normal variation in dietary patterns on daily glycemia within persons with T1DM, however, has not been investigated.

To examine the impact of daily variation in diet quality and assess the interaction between diet quality and exercise within individuals with T1DM, we used data from the Type 1 Diabetes Exercise Initiative (T1DEXI) cohort study [4]. Our objective for this analysis was to examine the impact of day-to-day variation in diet quality on acute measures of glycemia.

## Methods

The T1DEXI was a 4-wk at-home observational study of participants between 18 and 70 y of age who were diagnosed with T1D ≥2 y prior to starting the study [5]. Participants shared ≤4 wk of exercise and food intake data through the T1DEXI study smartphone application [6].

### Food photography and nutrient analysis

In addition to daily self-reported meal food intake information, which included meal descriptions and grams of carbohydrates, participants were asked to take photos of all meals and snacks using the T1Dexi App on the day of, and the day after, completing a study exercise video, for ≤12 total days. Participants were instructed to include a reference card (similar size to a driver’s license) in the image and to capture food photos at an arm’s distance away and a 45-degree angle. The T1Dexi application sent personalized text reminders to participants to take before and after photos of meals on the day of/day after the study exercise.

Food photos were analyzed by the Remote Food Photography Method to determine the nutrient intake of each meal [7]. A maximum of 8 d of food photos per participant were analyzed. Analyses of these photos per participant were prioritized to include food photo data days with CGM data, insulin data, and ≥2 meals with photos. If a participant missed taking a photo of a meal or snack, they could add text of meals consumed for which no photo was captured. Only 3.8% of the meals and snacks did not have a photo associated with them. A text description of a meal with a photo was used to provide additional information for these meals. A trained human rater using a computer-assisted approach to identify the foods and quantities consumed in the images and linked them to the Food and Nutrient Database for Dietary Studies [8] for nutrient analysis. Daily nutrient intake and consumption of servings of vegetables, fruits, grains, dairy, protein, fats, sugar, and sodium were calculated.

### Healthy Eating Index

Nutrition quality was assessed from daily total nutrient analysis using the Healthy Eating Index-2015 (HEI) formula [9], where a score has a range of 0–100 with higher scores representing a better-quality diet (meeting Dietary Guideline recommendations) [10]. It is important to note servings of foods are scaled per 1000 calories; HEI is a measure of overall diet quality relative to total caloric intake. HEI scores were calculated for each 24 h starting at 04:00 each morning. This hour cutoff was chosen due to it having the lowest number of recorded meals across all participants, suggesting that it was the most inclusive sleep period. The overall population means and median were similar and consistent with reported United States adult data [11]. We therefore categorized nutrition quality for each day as Low (HEI score ≤ 57) or High (HEI score > 57) based on the average HEI score for United States adults ages 19–59 y. Exploratory analyses also compared days with HEI scores in the first quartile compared days with HEI scores in the fourth quartile.

### Glycemic outcomes and insulin metrics

Glycemic outcomes were assessed from continuous glucose monitoring (CGM) data (Dexcom G6 CGM). CGM metrics were calculated for each day and then averaged across all high and low HEI days for each participant. CGM metrics included mean glucose (mg/dL), percent time in range (TIR) 70–180 mg/dL, percent time above range > 180 mg/dL, percent time > 250 mg/dL, percent time below range < 70 mg/dL, and percent time < 54 mg/dL. All CGM metrics were further tabulated by participants meeting and failing to meet an HbA1c goal of <7.0% at enrollment.

The percent time in the glycemic target range (TIR: 70–180 mg/dL) was also compared for exercise and sedentary days averaged across all high and low HEI days for each participant. An exercise day was defined as the succeeding 24 h after an exercise session, whereas a sedentary day was defined as any time >24 h from the last exercise session.

Participants continued their typical insulin regimens [multiple daily injections (MDI), pumps, or commercially approved hybrid closed-loop systems (HCL)] during the study. Insulin metrics analyzed included total daily dose, total daily basal dose, and total daily bolus dose (units/kg/d). For MDI users, insulin metrics were computed for days with a basal and bolus dose. For pump and HCL users, insulin metrics were computed for days with ≥18 h of pump data.

### Statistical analysis

For this analysis, we further restricted the days with HEI scores to those with ≥3 meals with food photos and ≥18 h of CGM data (Supplemental Figure 1). Additionally, only participants with ≥2 d of data in both HEI categories were included in this analysis. For each participant, CGM outcomes were averaged for all high HEI days and all low HEI days. This approach is analogous to calculating a participant’s CGM outcome after pooling all CGM data on low or high HEI days but gives equal weight to each day.

*P* values and confidence intervals were computed using a paired *t*-test. Due to a skewed distribution, the M-estimator was reported for percent time < 70 mg/dL, percent time < 54 mg/dL, and percent time > 250 mg/ dL, and the *P* values and confidence intervals were computed using a robust regression. A *P* value testing the interaction of the HEI group (low or high) and period status (exercise or sedentary) was performed for a percent time in the range of 70–180 mg/dL using a linear mixed effects model with HEI group, period status, and interaction as fixed effects and a random participant effect. Multiple comparisons were corrected for using the 2-stage false discovery rate correction procedure.

## Results

Of 561 participants in the T1DEXI study, 223 participants met the criteria for inclusion in these analyses (see flowchart of participant inclusion in Supplemental Figure 1). Baseline characteristics (Table 1) included a mean ± SD age of 37 ± 14 y (range, 19–68 y), T1DM duration of 18 ± 13 y, and a self-reported HbA1c at enrollment of 6.6% ± 0.7%. BMI (in kg/m^2^) was 25.1 ± 3.6 and 76% were female (*n* = 169). Insulin delivery method was 52% HCL, 42% pump, and 5% MDI.

**TABLE 1:** Participant characteristics.

	Overall (*N* = 223)
Age (y), mean ± SD (range)	37 ± 14 (19–68)
Gender – female *n* (%)	169 (76%)
HbA1c, mean ± SD	6.6 ± 0.7
	(49 ± 7.7)
<6.0% (<42 mmol/mol), *n* (%)	38 (17%)
6.0% to <6.5% (42 to <48 mmol/mol), *n* (%)	57 (26%)
6.5% to <7.0% (48 to <53 mmol/mol), *n* (%)	58 (26%)
7.0% to <7.5% (53 to <58 mmol/mol), *n* (%)	41 (18%)
7.5% to <8.0% (58 to <64 mmol/mol), *n* (%)	17 (8%)
≥8.0% (≥64 mmol/mol), *n* (%)	12 (5%)
Type 1 diabetes duration (y) *mean* ± *SD (range)*	18 ± 13 (2 to 62)
BMI (kg/m^2^), mean ± SD (range)	25.1 ± 3.6 (18.2 to 38.3)
Underweight (BMI <18.5 kg/m^2^), *n* (%)	1 (<1%)
Normal weight (BMI 18.5 to <25 kg/m^2^), *n* (%)	121 (54%)
Overweight (BMI 25.0 to <30 kg/m^2^), *n* (%)	78 (35%)
Obese (BMI ≥ 30 kg/m^2^), *n* (%)	23 (10%)
Race/ethnicity, *n* (%)	
White non-Hispanic	206 (92%)
Black non-Hispanic	2 (<1%)
Hispanic or Latino	4 (2%)
Asian	4 (2%)
American Indian/Alaskan Native	1 (<1%)
More than one race	5 (2%)
Unknown/not reported	1 (<1%)
Highest education level, *n* (%)	
<Bachelor’s	39 (17%)
Bachelor’s	99 (44%)
>Bachelor’s	84 (38%)
Unknown/not reported	1 (<1%)
Insulin method at enrollment, *n* (%)	
Multiple daily injections	12 (5%)
Non-HCL pump	94 (42%)
Hybrid closed loop	117 (52%)

Abbreviation: HCL, hybrid closed loop.

### Diet quality effects

**FIGURE 1:**
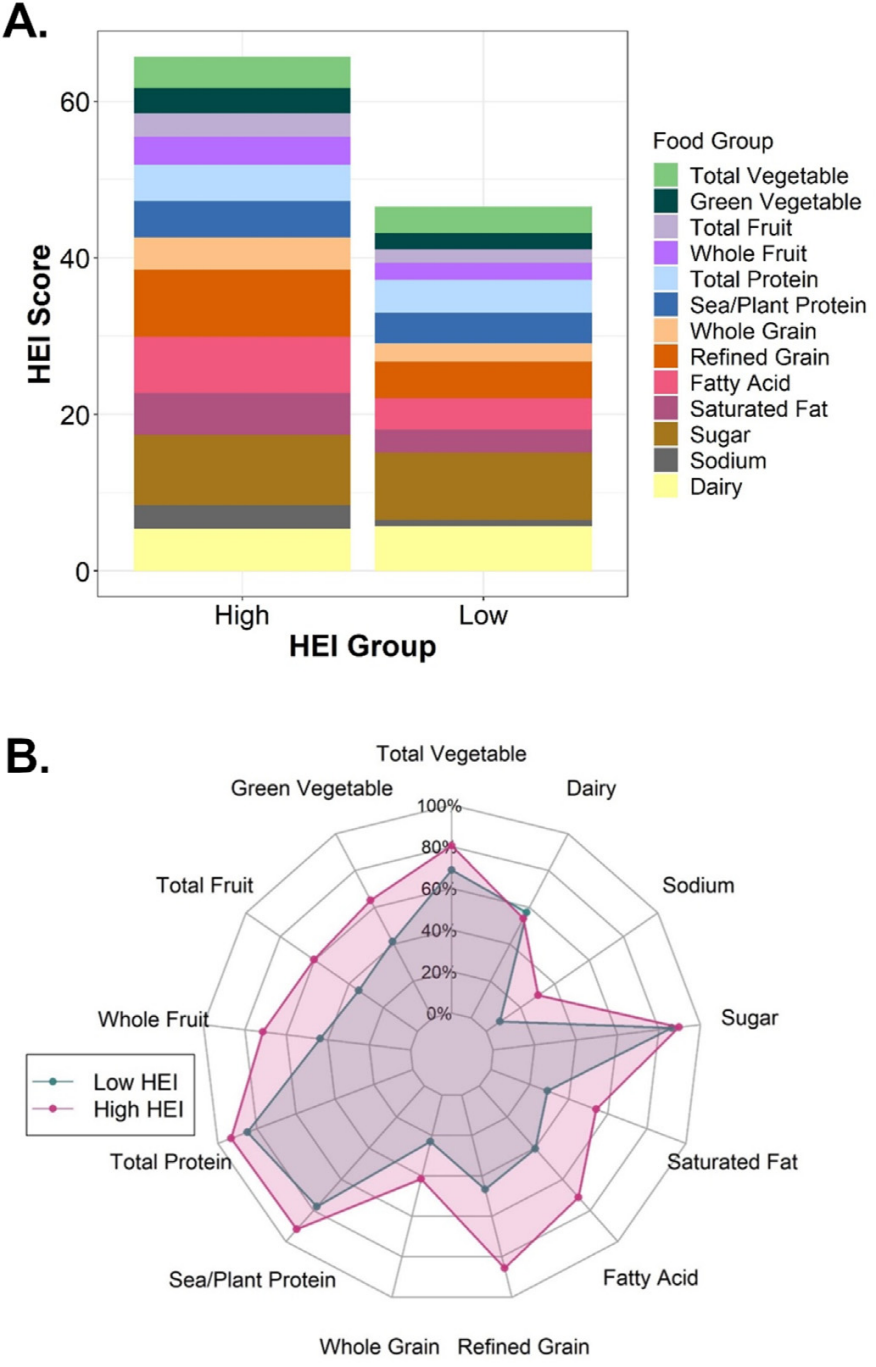
Stacked bar chart and spider plot of HEI score broken down by food group. (A) Stacked bar chart of HEI score. Each food group is represented by the raw HEI score resulting in the total HEI score being displayed as the height of the bar. Each food group has a maximum HEI value between 5 and 10. (B) Spider plot of HEI score. Each food group is represented as a percentage of total possible HEI value. HEI, Healthy Eating Index-2015; TIR, percent time in range.

The overall mean and median HEI scores were 56 for the 223 participants. There were 1182 high HEI days with a mean HEI score of 66 ± 4, and there were 1031 low HEI days with a mean HEI score of 47 ± 5. On high HEI days, mean daily intake was lower than low HEI days for saturated fats (22 g compared with 28 g), sodium (2562 mg compared with 3077 mg), and refined grains (2.7 ounce/1000 kcal compared with 4.7 ounce/1000 kcal), and higher for whole grains (1.22 ounce/1000 kcal compared with 0.65 ounce/1000 kcal). HEI scores for each of the 13 components on high and low HEI days are illustrated in Figure 1. Participants consumed less saturated fat and less refined grains, more polyunsaturated fat (PUFA), total fruit, and green vegetables during high HEI days. Categories such as sugar intake, seafood and plant protein, total protein, total vegetables, and dairy did not noticeably differ between high and low HEI days. Overall caloric intake on high HEI days was ∼100 kcal less than on low HEI days.

**FIGURE 2:**
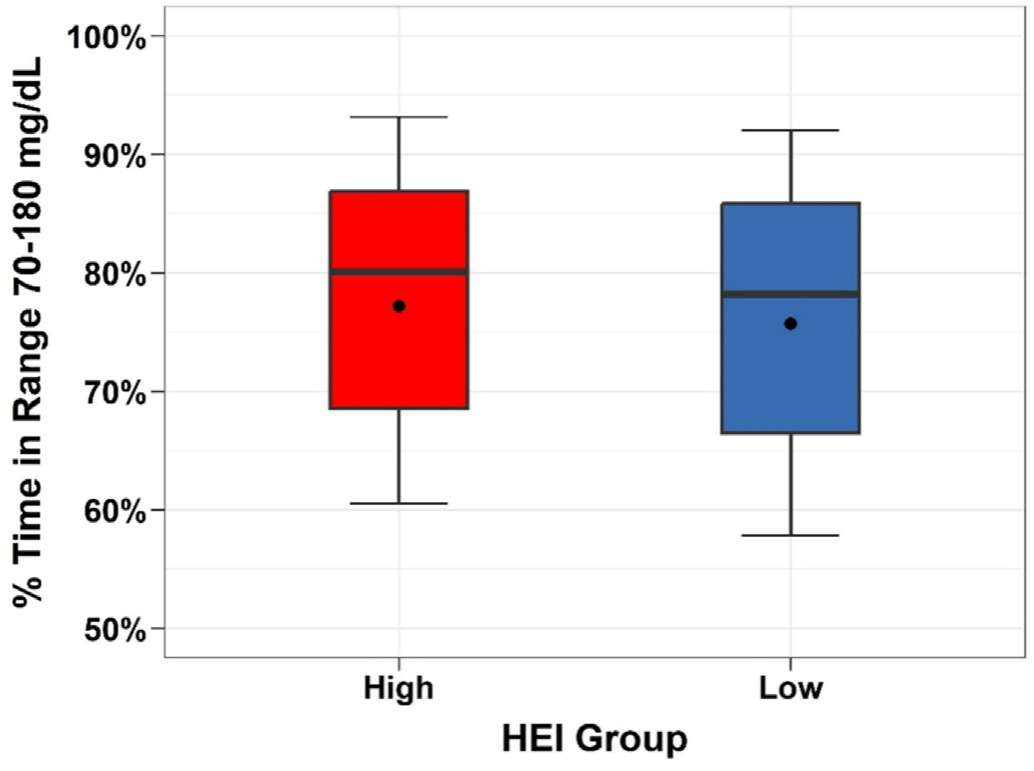
Boxplot of TIR on low HEI days and high HEI days (*N* = 223). HEI, Healthy Eating Index-2015; TIR, percent time in range.

The mean percent time in the range 70–180 mg/dL was 77.2% ± 14.1% on high HEI days compared with 75.7% ± 14.3% on low HEI days (adjusted group difference = 1.5%; 95% CI 0.3%–2.6%; *P* = 0.01; Table 2, Figure 2). Approximately 60% of all participants had an increased percent time in range on high HEI compared with low HEI days. Compared with low HEI days, high HEI days had slightly, but significantly, lower mean glucose (143 ± 22 compared with 145 ± 22 mg/dL, *P* = 0.02) and slightly lower mean percent time > 180 mg/dL (19.5% ± 14.2% compared with 21.2% ± 15.7%, *P* = 0.004). The percent time < 70 mg/dL and percent time < 54 mg/dL were similar for both groups. The total daily insulin dose was slightly lower on high HEI days compared with low HEI days (0.52 ± 0.18 compared with 0.54 ± 0.18 U/kg/d; *P* = 0.009). An exploratory analysis compared days with HEI in the fourth quartile against days with HEI in the first quartile and found similar results where the difference in mean TIR was 2.1% (Supplemental Figure 2).

**TABLE 2:** Within participant comparisons on outcome variables between days where diet quality was higher (high HEI) vs. lower (low HEI) (*N* = 223)

	High HEI Mean ± SD	Low HEI Mean ± SD	High vs. Low Mean difference (95% CI) (*P* value)^1^
Total HEI Score	66 ± 4	47 ± 5	
Mean glucose, mg/dL (mmol/L)	143 ± 22	145 ± 22	−2.2 (−4.0, −0.4) (0.02)
	(7.94 ± 1.22)	(8.05 ± 1.22)	
% Time in 70–180 mg/dL (3.89–9.99 mmol/L)	77.2% ± 14.1%	75.7% ± 14.3%	1.5% (0.3%, 2.6%) (0.01)
% Time >180 mg/dL (>9.99 mmol/L)	19.5% ± 14.2%	21.2% ± 14.7%	−1.7% (−2.9%, −0.6%) (0.004)
% Time <70 mg/dL (<3.89 mmol/L)	2.7% ± 2.9%	2.6% ± 2.6%	0.1% (−0.1%, 0.3%) (0.23)
% Time >250 mg/dL (>13.88 mmol/L)	3.1% ± 4.3%	3.7% ± 4.6%	−0.6% (−0.9%, −0.3%) (<0.001)
% Time <54 mg/dL (<3.00 mmol/L)	0.4% ± 0.6%	0.4% ± 0.6%	0.0% (−0.0%, 0.1%) (0.50)
Exercise duration (min/d)	59 ± 32	60 ± 36	
% Days with exercise	87% ± 21%	84% ± 19%	
% TIR exercise day^2^	77.6% ± 13.5%	76.3% ± 14.4%	
% TIR sedentary day^2^	76.8% ± 19.3%	73.1% ± 18.8%	
Meals eaten per day self-report	5.0 ± 1.5	4.8 ± 1.3	
Meals eaten per day by food photo	4.9 ± 1.4	4.7 ± 1.3	
Carbohydrates taken per day self-report (g)	139 ± 53	143 ± 57	
Carbohydrates taken per day food photo (g)	164 ± 54	171 ± 57	
Calories consumed per day (kcal)	1624 ± 427	1728 ± 482	
Total daily dose (U/kg/d)	0.52 ± 0.18	0.54 ± 0.18	−0.01 (−0.02, −0.00) (0.009)
Total bolus dose (U/kg/d)	0.26 ± 0.12	0.27 ± 0.13	
Total basal dose (U/kg/d)	0.27 ± 0.11	0.27 ± 0.10	

Abbreviations: CGM, continuous glucose monitoring; HEI, Healthy Eating Index-2015.

1 *P* values and confidence intervals calculated from a paired *t*-test. The % time <70 mg/dL, % time <54 mg/dL, and % time >250 mg/dL metrics were skewed, so the mean and standard deviation are derived from an M-estimator and the *P* value and confidence intervals calculated from a robust regression.

2 All CGM data 24 h after exercise were classified as an exercise period. If this period lasted ≥6 h during a day meeting all HEI criteria, it was included. Only participants with ≥1 high HEI exercise day and 1 low HEI exercise period were included when summarizing glycemia on exercise days (*N* = 222). Only participants with ≥1 high HEI sedentary day and 1 low HEI sedentary day were included when summarizing glycemia on sedentary days (*N* = 111).

The differences observed were not due to variable reporting or meal photo capture between high and low HEI days. Participants had a similar number of meals with photos during high HEI and the low HEI days. On average, there were 4.9 ± 1.4 meals with photos taken per day on high HEI days compared with 4.7 ± 1.3 meals with photos taken per day on low HEI days. Participants on average only had 3.8% of self-reported meals without a food photo on high HEI days and 4.5% of self-reported meals without a food photo on low HEI days. Regardless of overall diet quality, participants seemed to underestimate the total number of carbohydrates they consumed compared with the Remote Food Photography Method analysis as we have previously reported [6]. For high HEI days, the total carbohydrates consumed per day was 139 ± 53 g based on self-report compared with 164 ± 55 g from the meal photos. For low HEI days, the total carbohydrates consumed per day was 143 ± 57 g based on self-report compared with 171 ± 54 g from the meal photos.

### Diet quality and exercise interaction

Of all high HEI days in the analysis, 87% were captured during exercise days; only 13% of high HEI scores were captured during sedentary days. Similarly, of all the low HEI days, 84% were captured during exercise days; only 16% of low HEI scores were captured on sedentary days. This reflects the emphasis for participants to capture diet intake on structured exercise days during the study. Thus, the data analysis comparing high HEI compared with low HEI were more likely to be derived from comparisons on exercise days.

**FIGURE 3:**
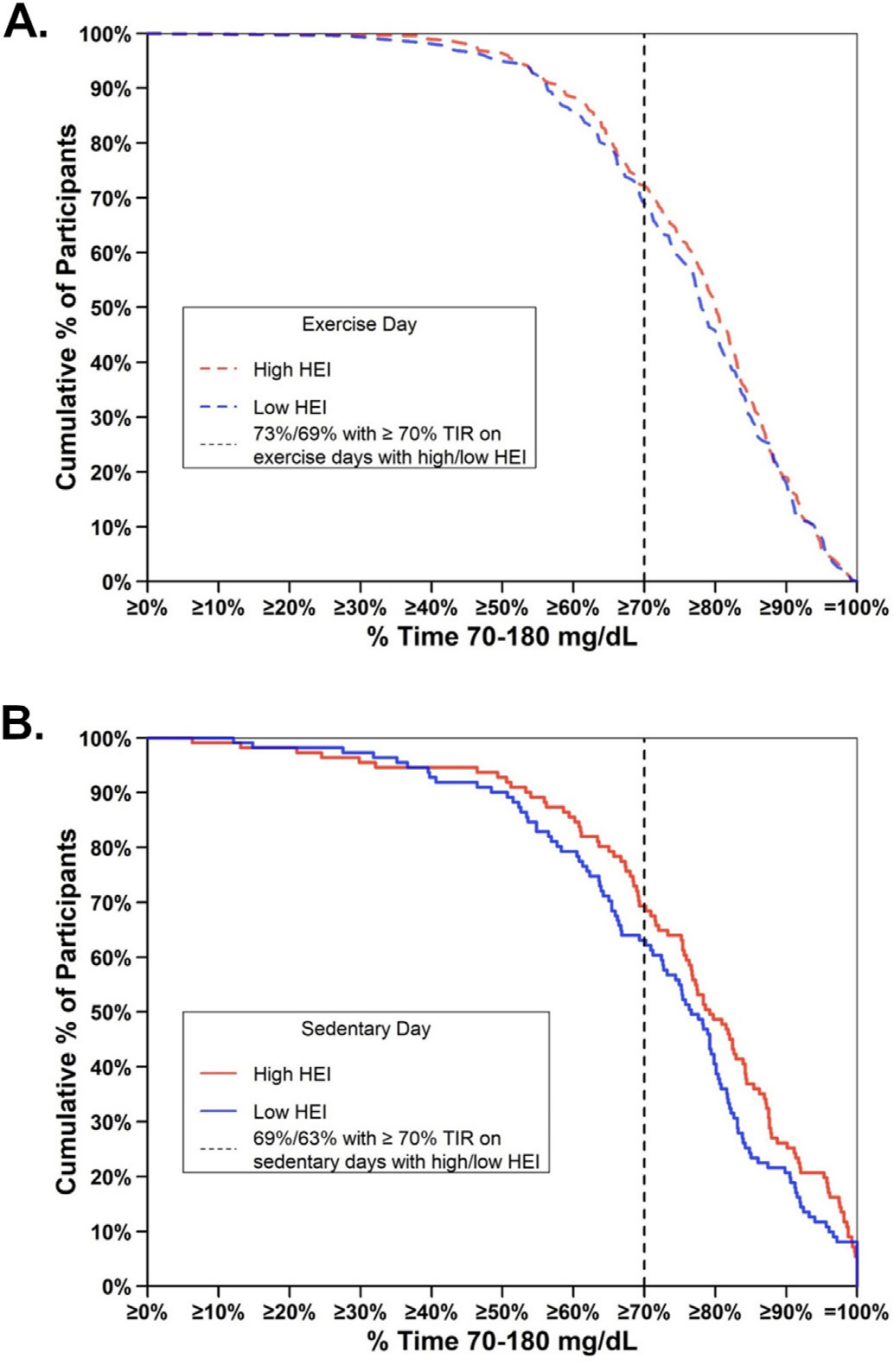
Participants’ TIR on low HEI days and high HEI days with exercise vs. sedentary days. Cumulative distribution of the TIR with high HEI (red) and low HEI (blue) during (A) exercise days and (B) sedentary days. HEI, Healthy Eating Index-2015; TIR, percent time in range.

On exercise days, the mean percent time in the range 70–180 mg/dL was 77.6% on high HEI days compared with 76.3% on low HEI days (Figure 3A). On sedentary days, the mean % time in the range 70–180 mg/dL was 76.8% on high HEI days compared with 73.1% on low HEI days (Figure 3B). Thus, the difference in mean percent time in the range 70–180 mg/dL on high HEI days compared with low HEI days was numerically greater on sedentary days, but the interaction of exercise status by HEI group did not reach statistical significance (*P* = 0.16). For participants with HbA1c < 7.0%, the mean percent time in the range 70–180 mg/dL was 81.3% on high HEI days compared with 79.3% on low HEI days; for participants with HbA1c ≥ 7.0%, the mean percent time in the range 70–180 mg/dL was 68.3% on high HEI days compared with 67.8% on low HEI days (Supplemental Table 1).

## Discussion

When participants with T1DM consumed a higher-quality diet, particularly higher in fruits, green vegetables, PUFA, and lower in saturated fat and refined grains, they had a clinically small but statistically significant increase in TIR, and lower mean 24 h glucose concentration and lower daily bolus insulin needs compared with days when the same participant consumed a lower-quality diet. Although statistically significant, these very small differences between high and low HEI days represent a clinically minor impact in overall glycemia, improving TIR by ∼15–20 min/d.

Total caloric and carbohydrate intake were similar between high and low HEI suggesting that the glycemic differences are related to the types of foods rather the quantity consumed. Our analysis is unique because we looked at day-to-day variation in diet quality within a participant and the acute impact of diet quality on glycemia during the concurrent 24-h period by CGM. The major advantage with this approach is that glycemic metrics are compared within participant and therefore the effect of diet quality can be assessed without the potential for participant confounding.

One of the more novel aspects of this study was our ability to look at the interaction of exercise and diet quality comparing glycemic metrics on exercise days compared with sedentary days. The difference in TIR between high/low HEI days was smaller on exercise days. Conversely, diet quality becomes a greater factor for improved glycemia on nonexercise days. However, the interaction between high/low HEI days and exercise compared with sedentary days on TIR was not significant in our model—possibly due to low power as there were fewer sedentary days because of the focus on collecting food photos on study exercise days in the study protocol. Our research group recently published the impact of exercise on total time in range. Exercise increased glycemic time in range by 6% compared with sedentary days [5]. In comparison, the magnitude of the impact of diet quality is smaller, 1.5%, representing an incremental improvement in this group of well-controlled individuals with T1DM. Consuming a high-quality diet is always beneficial, although our results may suggest it can also improve overall glycemic outcomes on days when a person with T1DM does not plan to exercise.

Several large cohort studies have investigated associations between diet patterns, including HEI score, and glycemia among individuals with T1DM, typically measured by HbA1c [1,2]. A higher diet quality was associated with better glycemic control and lower HbA1c in these cross-sectional analyses. For example, diets with a low glycemic index were associated with lower HbA1c, LDL, and triglycerides compared with high glycemic index diets [12,13]. Other studies used HEI-2005 to evaluate diet quality among youth and adolescents with T1DM. They reported associations between higher HEI scores with lower BMI and lower HbA1c; in particular, higher intake of plant-based foods and whole grains was associated with better glycemia [14,15]. Others noted youth with T1DM who consumed lower saturated fat and higher fiber had improved glycemic control [16]. Although all of these studies evaluated diet quality between groups of participants, our analysis looked at diet quality within individual participants and found that higher diet quality was associated with a modest but significant improvement in acute measures of glycemia.

Most cohort studies measure dietary intake at 1 or potentially 2 points in time. These measures of dietary intake likely do not capture inherent within-subject variability in dietary intake over a week or month. Within-subject variability upon test–retest studies of 24 h dietary recalls, or dietary records has historically been greater than between-subject variability indicating the high day-to-day variation of dietary intake among persons [3,17,18]. In this analysis, we have taken advantage of the within-subject variation in dietary patterns using food photo capture and compared that with the immediate glycemic response for a given study participant as measured by CGM. Participants did have variable dietary intake with HEI scores ranging from 16 to 96 during the study, with a mean and median HEI score of 56, consistent with the known variability in human diets. Given the acute nature of the outcome variable (CGM data), perhaps it is not surprising that differences in types of food consumed with a similar calorie and similar carbohydrate intake had a numerically small impact on glycemia.

In a subanalysis we looked at the impact of diet quality among participants with on-target or elevated HbA1c levels at study entry, hypothesizing that diet quality might have a larger impact among those with elevated HbA1c. However, we observed that for participants with good overall glycemic control, diet quality further improved total TIR more than for those participants with a higher HbA1c at study entry. The result was contrary to our initial hypothesis but suggests other factors that are associated with higher baseline HbA1c concentration, such as insulin delivery (basal or bolus dosing), carbohydrate-to-insulin ratios, or overweight/obesity may be of greater impact on daily glycemic TIR than overall diet quality among persons with an elevated HbA1c to start. These data also suggest that for such individuals, focused counseling on basic glycemic management may be of key import.

Our study has several limitations. Participant HbA1c was self-reported at enrollment; differences in the association between diet quality and glycemia in high and low HbA1c was based on categorizing participants from self-reported HbA1c. Some participants did not capture enough food photos or enough days of food photos to be included in this analysis. Despite the frequent text reminders via the T1Dexi application to capture food photos, complete dietary intake data remain extremely challenging to collect. The analysis excluded days with ≤3 meals with photos to ensure meals were properly recorded throughout the day, but results may not generalize to participants with fewer than 3 meals per day. The interaction of diet quality and structured exercise events was a particularly unique aspect of this analysis but is limited by the smaller data available on sedentary days. Moreover, study volunteers tended to be in good glycemic control, physically active, and predominately White non-Hispanic with underrepresentation of other ethnic and/or racial backgrounds.

There are several strengths of this analysis. We have a relatively large sample size, and we captured multiple days of food intake with food photography analytics and activity patterns using an activity monitor with concurrent measures of glycemia by CGM within participants. The interaction of diet quality and exercise is another unique aspect of the T1DEXI project.

In conclusion, consuming a higher-quality diet is associated with an incremental improvement in 24-h TIR among well-controlled adults with T1DM. Consistent with previous studies, diets lower in saturated fat and higher in fiber, fruits, green vegetables, and whole grains were associated with a small improvement in 24-h TIR metrics. Specifically, on nonexercise days, a healthy eating pattern was associated with ∼5% more of this cohort achieving the goal of >70% TIR. However, the overall glycemic impact of a single structured exercise session appears to have more beneficial impact on 24-h glucose TIR than dietary quality.

## Author contribution

The authors’ responsibilities were as follows – MBG, MCR, PC, RLG, SRP, PGJ, JRC, MAC, FJD, MRR, CKM: designed research; RLG, CKM: conducted research; MBG, MCM, MCR, PC, RLG: analyzed data; MBG, MCM: wrote the manuscript; MBG, CKM: shared primary responsibility for the final content; and all authors: read and approved the final manuscript.

### Conflict of interest

MBG, MCM, PC, RLG, FJD, and CKM report no conflicts of interest. MCR reports receiving consulting fees from the Jaeb Center for Health Research, Eli Lilly, Zealand Pharma, and Zucara Therapeutics; speaker fees from Sanofi Diabetes, Eli Lilly, Dexcom Canada, and Novo Nordisk; and stock options from Supersapiens and Zucara Therapeutics. SRP reports receiving grants from The Leona M. and Harry B. Helmsley Charitable Trust, the National Institutes of Health, and the Jaeb Center for Health Research; honorarium from the American Diabetes Association, outside the submitted work. PGJ reports receiving grants from the National Institutes of Health, The Leona M. and Harry B. Charitable Trust, the Juvenile Diabetes Research Foundation, Dexcom, and the Oregon Health & Science University Foundation; consultancy fees from CDISC; U.S. patents 62/352,939, 63/269,094, 62/944,287, 8810388, 9,480,418, 8,317,700, 61/570382, 8,810,388, 7,976,466, and 6,558,321; and reports stock options from Pacific Diabetes Technologies, outside submitted work. JRC reports receiving grants from the Juvenile Diabetes Research Foundation, the National Institutes of Health, Dexcom, and Medtronic; consultancy fees from Novo Nordisk, Insulet, and Zealand, outside the submitted work. MAC is the Chief Medical Officer of Glooko, Inc. and has received grants or contracts from Dexcom, Abbott Diabetes Care, the National Institutes of Health, the Juvenile Diabetes Research Foundation, the Emily Rosebud Foundation, Eli Lilly, Tolerion, and Garmin. MRR reports consultancy fees from Zealand Pharma.

### Funding

Research reported in this publication was supported by The Leona M. and Harry. B. Helmsley Charitable Trust. The content is solely the responsibility of the authors and does not necessarily represent the official views of The Leona M. and Harry B. Helmsley Charitable Trust. One of the author’s institutions (author CKM, Pennington Biomedical Research Center) is supported by NORC Center Grant P30 DK072476 entitled “Nutrition and Metabolic Health Through the Lifespan” sponsored by NIDDK and by grant U54 GM104940 from the National Institute of General Medical Sciences, which funds the Louisiana Clinical and Translational Science Center. Verily (South San Francisco, CA) provided the Study Watch at no cost. Dexcom provided continuous glucose monitors at a discounted rate.

### Data availability

The full data set is publicly available at https://doi.org/10.25934/PR00008428.
