# Supplementary material for: The Association Between Diet Quality and Glycemic Outcomes Among People with Type 1 Diabetes
Source: Curr Dev Nutr. 2024 Mar 26;8(4):102146. doi: 10.1016/j.cdnut.2024.102146 (PMC11024491; doi:10.1016/j.cdnut.2024.102146)

**The association between diet quality and glycemic outcomes among people with type 1 diabetes**

**First Author: Melanie B. Gillingham, PhD**

**Online Supplemental Material**

# Supplementary Figure S1. Participant Inclusion Flowchart.

**Initial Sample**

N Participants = 561

**Analysis Cohort**

N Participants = 223

N Days = 2,213

**<2 Low or <2 High HEI days**

N Participants Excluded = 163

**<18 hours of CGM data**

N Participants Excluded = 64

**<3 meals per day**

N Participants Excluded = 111

N Participants = 386

N Days = 3,284

N Participants = 497

N Days = 12,918

# Supplemental Table S1. Data summarized between participants with high vs. low HbA1c on days when participants ate higher vs. lower quality diets.

|  | **HbA1c <7.0% (N=153)** | | **HbA1c ≥7.0% (N=70)** | |
| --- | --- | --- | --- | --- |
|  | **High HEI**  **Mean ± SD** | **Low HEI**  **Mean ± SD** | **High HEI**  **Mean ± SD** | **Low HEI**  **Mean ± SD** |
| Total HEI Score | 66 ± 5 | 47 ± 4 | 65 ± 4 | 45 ± 5 |
| Mean Glucose mg/dL | 136.2 ± 16.1 | 139.1 ± 18.9 | 156.6 ± 26.4 | 157.1 ± 24.5 |
| % Time in 70-180 mg/dL (TIR) | 81.3% ± 10.8% | 79.3% ± 12.4% | 68.3% ± 16.4% | 67.8% ± 15.2% |
| % Time >180 mg/dL | 15.3% ± 10.4% | 17.6% ± 12.7% | 28.6% ± 17.0% | 29.2% ± 15.8% |
| % Time <70 mg/dL ^a^ | 2.9% ± 2.7% | 2.7% ± 2.7% | 2.3% ± 2.9% | 2.5% ± 2.3% |
| % Time >250 mg/dL ^a^ | 0.0% ± 0.0% | 0.3% ± 0.4% | 1.6% ± 2.4% | 2.0% ± 3.0% |
| % Time <54 mg/dL ^a^ | 0.4% ± 0.6% | 0.4% ± 0.6% | 0.3% ± 0.5% | 0.4% ± 0.6% |
| Exercise Duration (min/day) | 59 ± 33 | 58 ± 38 | 60 ± 28 | 64 ± 30 |
| % Days with Exercise | 86% ± 21% | 84% ± 19% | 88% ± 22% | 85% ± 21% |
| % TIR Exercise Day ^b^ | 81.0% ± 11.4% | 79.6% ± 13.2% | 70.0% ± 14.6% | 69.0% ± 14.3% |
| % TIR Sedentary Day ^b^ | 80.8% ± 14.3% | 75.1% ± 17.7% | 66.3% ± 26.0% | 67.8% ± 20.8% |
| Meals Eaten Per Day Self-Report | 5.1 ± 1.6 | 4.9 ± 1.4 | 4.6 ± 1.1 | 4.5 ± 1.3 |
| Meals Eaten Per Day by Food Photo | 5.1 ± 1.6 | 4.8 ± 1.4 | 4.6 ± 1.1 | 4.5 ± 1.3 |
| Carbs Taken Per Day Self-Report (g) | 145 ± 56 | 148 ± 61 | 125 ± 43 | 131 ± 46 |
| Carbs Taken Per Day by Food Photo (g) | 166 ± 56 | 172 ± 57 | 160 ± 52 | 170 ± 58 |
| Calories Consumed Per Day (kCal) | 1658 ± 422 | 1776 ± 493 | 1549 ± 431 | 1625 ± 444 |
| Total Daily Dose (U/kg/day) | 0.53 ± 0.18 | 0.54 ± 0.18 | 0.51 ± 0.17 | 0.53 ± 0.18 |
| Total Bolus Dose (U/kg/day) | 0.26 ± 0.11 | 0.27 ± 0.13 | 0.24 ± 0.12 | 0.26 ± 0.13 |
| Total Basal Dose (U/kg/day) | 0.27 ± 0.12 | 0.26 ± 0.10 | 0.27 ± 0.09 | 0.28 ± 0.09 |

a – The % time <70 mg/dL, % time <54 mg/dL, and % time >250 mg/dL metrics were skewed, so the mean and standard deviation are derived from an M-estimator.

b – All CGM data 24 hours after exercise was classified as an exercise period. If this period lasted at least 6 hours during a day meeting all HEI criteria, it was included. Only participants with at least 1 high HEI exercise day and 1 low HEI exercise day were included when summarizing glycemia on exercise days (N=153 for HbA1c <7.0%, N=69 for HbA1c ≥7.0%). Only participants with at least 1 high HEI sedentary day and 1 low HEI sedentary day were included when summarizing glycemia on sedentary days (N=70 for HbA1c <7.0%, N=31 for HbA1c ≥7.0%).

# Supplementary Figure S2. Participants’ TIR on Days with Low HEI (First Quartile), Medium HEI (Second and Third Quartiles), and High HEI (Fourth Quartile). Participants needed to have at least two day in each HEI group (N=170) to be included in this analysis.


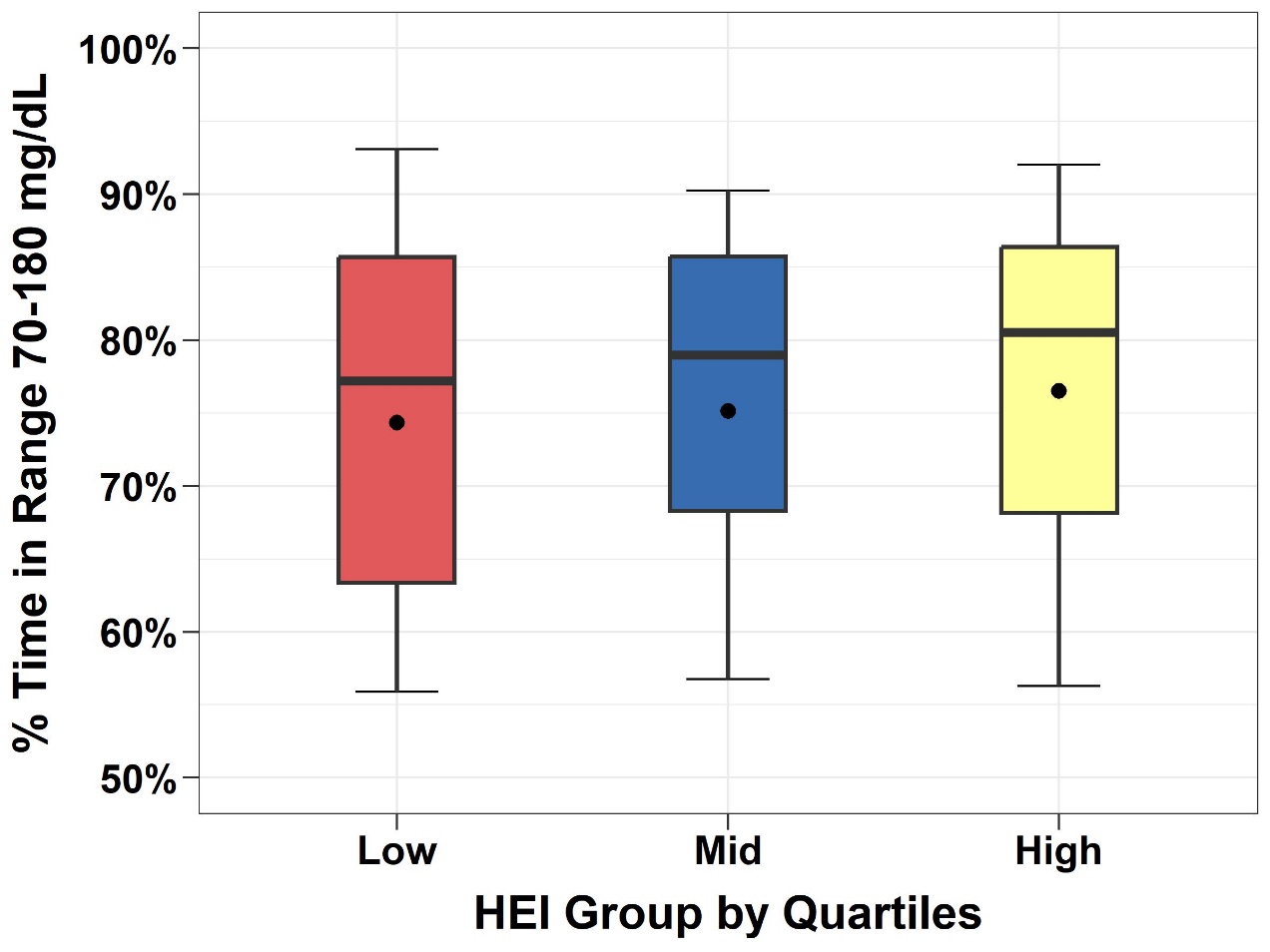

Supplement: Multimedia component 1 [file mmc1.docx]
